# Arguments from Developmental Order

**DOI:** 10.3389/fpsyg.2016.00751

**Published:** 2016-05-20

**Authors:** Richard Stöckle-Schobel

**Affiliations:** Chair of Philosophy of Language and Cognition, Department of Philosophy II, Ruhr University of BochumBochum, Germany

**Keywords:** development, developmental order, inference to the best explanation, philosophy of science, argumentation, conditional reasoning, theories of concepts, developmental psychology

## Abstract

In this article^1^, I investigate a special type of argument regarding the role of development in theorizing about psychological processes and cognitive capacities. Among the issues that developmental psychologists study, discovering the ontogenetic trajectory of mechanisms or capacities underpinning our cognitive functions ranks highly. The order in which functions are developed or capacities are acquired is a matter of debate between competing psychological theories, and also philosophical conceptions of the mind – getting the role and the significance of the different steps in this order right could be seen as an important virtue of such theories. Thus, a special kind of strategy in arguments between competing philosophical or psychological theories is using developmental order in arguing for or against a given psychological claim. In this article, I will introduce an analysis of arguments from developmental order, which come in two general types: arguments emphasizing the importance of the early cognitive processes and arguments emphasizing the late cognitive processes. I will discuss their role in one of the central tools for evaluating scientific theories, namely in making inferences to the best explanation. I will argue that appeal to developmental order is, by itself, an insufficient criterion for theory choice and has to be part of an argument based on other core explanatory or empirical virtues. I will end by proposing a more concerted study of philosophical issues concerning (cognitive) development, and I will present some topics that also pertain to a full-fledged ‘philosophy of development.’

## Introduction

A fundamental topic in the philosophy of psychology is the role of development in theorizing about psychological processes and cognitive capacities. Developmental psychologists track the trajectories of cognition on several levels and for a wide variety of cognitive functions. They establish an order in which functions are developed or capacities are acquired. When comparing alternative theories of the mind, the significance of the different steps in this order can be debated.

A special kind of strategy in arguments between competing philosophical or psychological theories is using developmental order in arguing for or against a given psychological claim. In this article, I will introduce an analysis of arguments from developmental order and I will discuss their role in making inferences to the best explanation. I will argue that appeal to developmental order is, by itself, an insufficient criterion for theory choice and has to be part of an argument based on other core explanatory or empirical virtues.

## Developmental Order

I want to begin by considering what is at stake when talking about the developmental order of mental processes. There are developmental trajectories for a large number of mental processes *M.* We can observe that the functioning of *M* changes over the human lifespan. Suppose that there is empirical evidence that children use process *P_1_* for a mental process *M*. During cognitive development, further processes *P_2_, P_3_*, and *P_4_* start to replace or to support *P_1_*. In adulthood, the set of mental capacities used for *M* might contain *P_3_* and *P_4_*, or evolved forms of *P_1_* and *P_4_*, just *P_4_*, or a different combination of processes. As a simplified example of a trajectory and processes, consider number cognition. The trajectory from a very limited understanding of quantities in infancy to the ability to use rational numbers in adolescence is explained by appeal to three sets of cognitive abilities, according to Carey (2009): first, there are mechanisms for differentiating small quantities, and for roughly computing large differences between sets of things. Next, with learning the number words, the ability to count and to use natural numbers develops. Third, with learning even more mathematical theory, we gain the ability to use division and to thus use rational numbers.

Even while agreeing with Thelen and Smith (1994) that development is not a single, linear process and that setbacks and various different ‘architectures’ can lead to a fully developed cognitive system, I think it is fair to assume that, for any given cognitive process, there are several theories with at least some empirical support at several points in ontogeny. Some of these are bound to be in conflict about the proper interpretation of the data and about the correct analysis of the developmental trajectory.

In the cognitive sciences of development, we can thus ask: Which mechanism or which process (e.g., P_1_, P_2_,…) is *fundamental* for *M* in human cognition? We can use *conceptual thinking* as an example for *M*; which type of concepts, or which conceptual structure do we find in human development? There are several theoretical frameworks for the study of concepts. The most widely discussed ones are Prototype Theory (PT; Rosch and Mervis, 1975; Rosch, 1978), Exemplar Theory (ET; Medin and Smith, 1981; Nosofsky, 2014), Theory Theory (TT; Keil, 1989; Gopnik and Meltzoff, 1998), and Neo-Empiricist Theories (NET; Prinz, 2002). They all differ in their definition of what concepts are, and in their explanation of how concepts play the role of constituents of thought, broadly construed. As such, each of them is bound to tell a different developmental story, with its own order of processes *P_1PT_*, … *P_iPT_*, etc.

This wide range of alternative frameworks has led some researchers to consider the possibility of a pluralism of types of concepts (Gelman, 2004; Weiskopf, 2009; Rice, 2016). Machery (2009) uses these pluralist considerations to argue for the elimination of *concept* as a term of theoretical significance in the cognitive sciences. For present purposes, I want to consider how the potential plurality of types of concepts might be debated in developmental terms. After all, if there were a good argument from developmental order, showing that one type of concept indeed is developmentally more fundamental than the others, one would have a reason to doubt Machery’s eliminativist argument.

## Priority of the Early Process: Dev_Early

The first argumentative strategy when discussing a given developmental order of psychological processes is what I call the *Dev_Early* view. One of its instantiations is to hold that the developmentally early process *P_1_* is the best model for *M*; other construals are in principle consistent with the view, e.g., that *P_1_* and *P_2_* give the preferable explanation (either in conjunction, or as alternatives that the cognitive system weighs contextually). A theory of *M* should thus focus on the first processes for *M* in their models. The initial processes, the argument goes, is not replaced by later developments; rather, more elaborate versions of *P_1_* play its role in adults, or *P_1_* by itself would be sufficient for performing *P_4_*’s role in adult cognition. Without *P_1_* as the starting point, *M* might have developed in a radically different way, or it would not have been possible to achieve this mental capacity.

Using the example of concepts, one possible position to hold is the following: When looking at the developmental data available, one type of concept emerges as the best explanation of infant performance. For example, Yermolayeva and Rakison (2009) cite evidence from face recognition studies that infants first use exemplars, and only later begin using frequency information (prototypes) and, even later, causal principles and rules (theories).^2^ If the experiments only revealed a reliance on other processes later in development, one might argue that they cannot be fundamental for conceptual thinking. Thus, the developmental order of acquisition might be used as a reason to argue for a theoretical position.

## Priority of the Late Process: Dev_Late

However, one can also adopt the *Dev_Late* position when arguing about developmental orders. This would take the form of arguing that explaining *M* is best achieved by appeal to the developmentally late process *P_4_*, or again to a combination of late processes *P_3_* and *P_4_*, as above. Since it is the mature form of the process, *P_4_* is the best foundation for a theory of *M*. Among its advantages, one might find its better integration with other mental processes. For example, if *P_1_* were a simple form of associative thought, and *P_4_* were a sophisticated reasoning heuristic, the latter process’s integration with background knowledge and broad applicability would speak for privileging it. Also, one could make a *normative* argument for *P_4_.* If done *right, M* works by P_4_, i.e., it is the best tool for solving the given cognitive problem, or for performing the given task. Thus, one should base one’s theory on the assumption that the late process is fundamental or central.

Using the example of concepts again, one line of argument might be that there is a higher cognitive power in having a diverse range of kinds of concept available and that there is no explanatory gain in regarding any one of them as primary. Hybrid Theorists such as Rice (2016) argue for a multitude of types of concepts that rely on different kinds of information, but which are able to fulfill similar cognitive roles. Just because one type is ontogenetically prior, one should not privilege it above the other types; developmental *superiority* trumps priority, as it were.

## A Second Example: Conditional Reasoning

Similar forms of argumentation surface in other areas of research. Consider the development of conditional reasoning as another example. Again, there are several theories that attempt to explain the way humans reason with conditionals. The three most notable groups of theories are Mental Models accounts (Johnson-Laird and Byrne, 2002; Thompson and Byrne, 2002; Byrne and Johnson-Laird, 2009; Johnson-Laird, 2010), Suppositional accounts (Edgington, 1995; Bennett, 2003; Evans and Over, 2004; Oaksford and Chater, 2007), and Mental Logic accounts (Rips, 1994; Braine and O’Brien, 1998). In several recent studies, Gauffroy and Barrouillet (2014), and Barrouillet and Gauffroy (2015) have proposed a developmental trajectory for conditional reasoning. They advocate a modified Mental Models account as fundamental for understanding conditionals (cf. Barrouillet and Gauffroy, 2015, p. 34). Barrouillet and Gauffroy (2015) propose that the interpretation of conditionals, specifically probabilistic conditionals, evolves from an initial incomplete mental model (which treats a conditional as equivalent to a conjunction) to a more fleshed-out mental model that corresponds to a defective biconditional interpretation to a model that corresponds with the Suppositional Theory’s strategy for evaluating conditionals (i.e., some variant of applying the Ramsey Test, cf. Edgington, 1995; Willer, 2010). Assuming that these results are robust, one can once again use an argument from developmental order in either direction:

*Dev_Early – Cond*: Interpreting the late developmental achievements as extensions of the developmental early capacity.

Thereby, one would focus on the developmental stability – some adult participants in their experiments still use the ‘conjunctive’ conditional rather than the ‘suppositional’ conditional – and on the continuities in reasoning – one would be able to justify a re-interpretation of the ‘suppositional’ responses as more refined mental-model-responses (cf. Barrouillet and Gauffroy, 2015, p. 34). The late developmental achievements found in adult reasoners are taken to speak against the Suppositional account, as that explanation is supposedly incompatible with the earlier developmental data (the reliance on a conjunctive understanding of the conditional).

*Dev_Late – Cond*: Appealing to the supposed normative correctness of the theory that best corresponds to the late developmental achievements, and diminishing the role of earlier developmental capacities by highlighting the disadvantages of the theories that champion them as the best normative theories.

A typical explanation of the ‘normatively incorrect’ performance in younger ages would be to appeal to factors that inhibit the employment of the ‘normatively correct’ strategy – in analogy with examples like the inhibitors (weight gain in the legs) that prevent infants from making stepping motions after 2 months of age (cf. Thelen and Smith, 1994, p. 11f.), or by appeal to the performance-competence distinction (cf. Chomsky, 1965; Le Corre et al., 2006). Furthermore, potential proponents of the *Dev_Late – Cond* view – proponents of the Suppositional Theory – could point to the high numbers of responses that uniquely fit with their predictions, both in their own studies (cf. Over et al., 2007) and in Gauffroy and Barrouillet’s (2014) experiments.

With these descriptions and examples in mind, I would like to turn to the question of the explanatory status of arguments from developmental order.

## Explanatory Considerations

Prima facie, both kinds of argument have virtues that we look for in psychological explanations. After all, they rely on a potentially wide range of empirical evidence and they are attempts at providing a bigger-picture view of a research topic. I want to consider whether appeals to the early or late temporal position of a cognitive process relates to special (or especially important) virtues of a theory. When adjudicating between different theories, would an Inference to the Best Explanation (IBE) categorically favor *Late_Dev* over *Early_Dev*, or the other way around?

Inference to the Best Explanation is a method for evaluating scientific theories. When considering a body of empirical data and several rival theories that attempt to make sense of the data, we should choose the theory that gives the best explanation of the observations, assuming that the theory itself conforms to some standards of adequacy (cf. Lipton, 2004). In general, Lipton proposes that the aim of a theory should be to give a *lovely* explanation – an explanation which has many of theoretical virtues is supposed to be a good explanation. Lovely explanations, in turn, are more likely to be conducive to truth. So, the loveliest explanations are supposed to be coextensive with the likeliest explanations (cf. Lipton, 2004, p. 61).

The typical virtues that IBEs rely on are theoretical virtues such as “scope, precision, mechanism, unification (…) [or] simplicity” (Lipton, 2000, p. 187), but there are also empirical virtues to be considered, such as fit of a theory with empirical data or superior ability to make empirical predictions (cf. Van Fraassen, 1977; Ruhmkorff, 2005).

Inferences to the best explanation, however, are not a miracle cure in disputes between competing theories. As Sprevak (2010) points out, arguing for or against a theory by appeal to an IBE is difficult at best and unjustified at worst, since “IBE is highly sensitive to the competitive context” (Sprevak, 2010, p. 354).

Based on a comparison with these main types of virtues on which IBEs are typically based, I want to argue that developmental order is not a decisive criterion for favoring one theory over another; both argumentative strategies can only succeed in combination with an appeal to other theoretical or empirical virtues. The argument for this position is as follows.

A given theory *A* that can adequately explain a large part of the empirical results from developmental research and uses this for a *Dev_Early* argument is *ceteris paribus* better than an alternative theory *B* which has all of *A*’s virtues except the fit with developmental data (with or without an argument by developmental order). However, a comparison between theory *A* and a rival theory *C*, which is equally theoretically virtuous and developmentally informed, but gives a *Dev_Late* argument supporting its differing hypotheses, is a much less clear-cut affair. Just because a mental process *P_1_* is used early in development, it isn’t necessarily a good candidate as the model for how we should explain human cognition with regard to *P_1_*’s functional domain. It only becomes a good candidate if we find additional reasons to regard it as such, such as continuity throughout the lifespan, or evolutionary continuity with other species (if for example great apes also used *P_1_*). Equally, a given mental process *P_4_* is not superior just because it is the last addition to the ‘mental toolbox’ for solving a cognitive problem. Rather, it becomes the superior candidate by, for instance, being the most reliable mechanism for solving the problem, or by being the most flexible mechanism. Our choice of proposed mechanism to solve cognitive problems should thus depend on the kind of theory we want to advocate and its fit with the available data, in conjunction with the ability to generate good predictions.

To illustrate this, consider the example from the conditional reasoning literature. If my position holds, neither the Mental Models theorist nor the Suppositional theorist can cash in on their *Dev_Early/Late* argument by itself. The success of their arguments rather depends on whether one finds that the developmental continuity of conditional reasoning capacities is a bigger virtue than the better compatibility with adult reasoning results, or *vice versa*. Depending on such choices of priorities in theoretical virtues, and further depending on an analysis of the explanatory virtues of the competing theories, one can then go on and endorse the theory that indeed gives the best explanation.

Furthermore, deliberation about theory choice should not ultimately be decided by appeal to developmental order, but rather by a broad consideration of the fit between theoretical explanations and empirical evidence. Before we can endorse a *Dev_Early* or a *Dev_Late* argument, we need to think through deeper philosophical issues, such as the ones I raised above as justifications for taking either stance; for example, without an account of what we take the normative aim of a given mental process to be, without an understanding of the role of the earliest cognitive capacities (and indeed, without a stance on innateness and on cognitive change), or indeed without an account of the evolutionary role of a cognitive function (among, of course, several other issues), we risk telling ‘just-so stories’ when appealing to developmental order.

I would like to emphasize that this is not meant to diminish the role of experimental studies of the developmental trajectories of our cognitive processes. Developmental psychology has made tremendous progress in terms of methodology and breadth of investigation in the last decades, and we can only expect to gain a deeper understanding of human psychology by continuing our present efforts. However, I want to urge caution and care in theorizing about these empirical results.

## Outlook: A Philosophy of Development?

I would like to finish this short investigation by linking it to a broader philosophical project of thinking about cognitive development. Admittedly, developmental research has grappled with questions related to the issues identified above for quite some time. To wit, the issue of (dis)continuity in conceptual change covers a similar ground – are our concepts continuous throughout the lifespan, or are there radical changes in our conceptual abilities that make earlier and later concepts incommensurable (cf. Keil, 1981; Carey, 2009)?

Another related issue concerns the study of developmental trajectories more generally. As Adolph et al. (2008) observe, the shape of such trajectories crucially depends on the choice of measuring intervals. To get a clearer understanding of the onset time of a newly developing capacity, often a fine grain of measurement is necessary.

A further investigation of questions about development will need to connect the issues raised in this paper to issues in ethology, most notably to Tinbergen’s four questions (Tinbergen, 1963) and to questions regarding the relations between phylogeny and ontogeny on several theoretical levels (Stamps, 2003; Bateson and Laland, 2013).

In line with the above considerations from the philosophy of science, I would like to offer the present paper as a call for more work on the philosophical questions regarding cognitive development. At the intersection of psychology, philosophy of science, and philosophy of mind, there are many open questions that should be addressed by a ‘philosophy of development.’ Thinking about the limitations of arguing for a theory by appeal to development is only one of many possible starting points.

## Author Contributions

The author confirms being the sole contributor of this work and approved it for publication.

## Conflict of Interest Statement

The author declares that the research was conducted in the absence of any commercial or financial relationships that could be construed as a potential conflict of interest.

## Footnotes

2 Note that Murphy (2002) interprets the infant research to show a slight preference for prototypes instead of exemplars, but for present expository purposes, I regard Yermolayeva and Rakison (2009)’s position more as proof-of-concept rather than as an established possibility.
